# Detection of malingering: psychometric evaluation of the Chinese version of the structured interview of reported symptoms-2

**DOI:** 10.1186/1471-244X-13-254

**Published:** 2013-10-09

**Authors:** Chang Liu, Zhening Liu, Helen FK Chiu, Tam Wai-Cheong Carl, Huiran Zhang, Peng Wang, Guowei Wu, Tumbewene E Mwansisya, Longlong Cao, Aimin Hu, Yu Wang, Zhimin Xue

**Affiliations:** 1 Mental Health Institute of The Second Xiangya Hospital, Key Laboratory of Psychiatry and Mental Health of Hunan Province, Central South University, Changsha, 410011, Hunan, China; 2 Department of Psychiatry, The Chinese University of Hong Kong, Hong Kong, SAR, China; 3 Department of Psychology, Chung Yuan Christian University Chung Li, Taiwan 32023, China; 4 Department of Psychiatry, The Second Affiliated Hospital, Xin Jiang Medical University, Urumqi 830061, China

**Keywords:** Malingering, The Chinese version of the structured interview of reported symptoms-2, Reliability, Validity

## Abstract

**Background:**

Malingering detection has emerged as an important issue in clinical and forensic settings. The Structured Interview of Reported Symptoms-2 (SIRS-2) was designed to assess the feigned symptoms in both clinical and non-clinical subjects. The aim of the study was to examine the reliability and validity of the Chinese version of this scale.

**Methods:**

Two studies were conducted to evaluate the reliability and validity of the Chinese Version of SIRS-2. In Study one, with a simulation design, the subjects included a. 40 students asked to simulate symptoms of mental illness; b. 40 general psychiatric inpatients and c. 40 students asked to reply to questions honestly. Scales scores for feigning symptoms among three groups were carried out for discriminant validity of the Chinese Version of SIRS-2. Minnesota Multiphasic Personality Inventory-2(MMPI-2) was administered in 80 undergraduate students. In Study two, with a known-groups comparison design, scales scores for feigning symptoms were compared between 20 suspected malingerers and 80 psychiatric outpatients from two forensic centers using the Chinese Version of SIRS-2.

**Results:**

The Chinese Version of SIRS-2 demonstrated satisfactory internal consistency in both study one and two. In study one, criterion validity of this scale was supported by its significantly positive correlation with the MMPI-2 (r = 0.282 ~ 0.481 for Infrequency), and by its significantly negative correlation with the MMPI-2 (r = -0.255 ~ -0.519 for Lie and -0.205 ~ 0.391 for Correction). Scores of 10 out of 13 subscales of the Chinese Version of SIRS-2 for simulators were significantly higher than scores of honest students and general psychiatric patients. In study two, the mean scores of the Chinese Version of 13 subscales for suspected malingerers were significantly higher than those of psychiatric outpatients. For discriminant validity, it yielded a large effect size (d = 1.80) for the comparison of the participant groups in study one and two. Moreover, the sensitivity (proportion of malingerers accurately identified by the measure) and specificity (proportion of people accurately classified as responding honestly) of the Chinese version of SIRS-2 in the detection of malingering in these two studies are acceptable.

**Conclusions:**

The Chinese version of the SIRS-2 has good psychometric properties and is a valid and reliable tool for detection of malingering in Chinese populations.

## Background

According to the DSM-IV-TR, malingering is used to denote “the intentional production of false or grossly exaggerated physical or psychological symptoms, motivated by external incentives such as avoiding military duty, avoiding work, obtaining financial compensation, evading criminal prosecution, or obtaining drugs”. The surveys for forensic and non-forensic referrals yielded the mean percentages of about 15% and about 7% respectively of malingering [1]. Chiang et al. [2] found that 9.1% of the people in a sample of draftees seeking psychiatric re-evaluation were suspected to be malingerers in Taiwan. The phenomenon of malingering creates social compensation problems and wasting of medical resources. However, detection of malingering is not easy. There were some screening indices in DSM-IV-TR, but these have not been rigorously tested [3]. Available data suggest that these indices may produce unacceptably high false-positives in the neighborhood of 80% [4]. Several psychological tests have been developed in clinical settings, such as the MMPI-2, Personality Assessment Inventory (PAI), and Millon Clinical Multiaxial Inventory-III (MCMI-III) [5,6]. However, these measures were not specifically designed to detect malingering.

In China, there are very few studies addressing the issue of malingering and its detection. For example, Liu [7] described the assessment of malingering in clinical settings, while Yang [8] reviewed the psychopathology and diagnosis of malingering. However, very few studies have examined malingering with a special and standardized measure in Chinese populations.

The Structured Interview of Reported Symptoms -2 (SIRS-2) is one of the most well-known psychological instruments designed to assess malingering [9]. Unlike the MMPI, the SIRS has been developed specifically to assess whether an examinee is feigning psychological symptoms. The SIRS-2 is a 172-item, interviewer-administered rating scale that relies on empirically-based strategies to assess malingering [9]. The SIRS-2 has been validated for use in clinical and non-clinical samples [9]. The SIRS-2 is widely used for the identification of malingering in forensic psychiatry [10], and it has been validated for the detection of feigning specific disorders or cognitive deficits among psychiatric populations and adolescent offenders [11-13]. However, in some cases, the SIRS-2 might misclassify the examinee, which will limit the SIRS-2 applicability. For example, traumatized inpatients with a broad array of presenting symptoms, especially those who report childhood trauma and dissociative symptoms have been misclassified [11].

The SIRS-2 [14] has been found to have moderately high internal consistency with Cronbach ’s alphas coefficient for three subscales (primary scales ranging from .77 to .92; supplementary scales ranging from .75 to .82). The criterion validity, predictive validity and test-retest reliability of SIRS-2 have been established as well [15]. Moreover, the SIRS-2 is one of the few malingering assessment measures to have the discriminant validity for malingering in clinical and non-clinical samples. Specifically, honest samples were found to score higher than malingerer samples in both simulation and known-groups comparison with large effect sizes to discriminate suspected malingerers from controls (mean d = 1.74) [16].

The literature indicates that the SIRS-2 is one of the best instruments for the detection of malingering to date. However, there is no reliable and valid Chinese instrument for malingering detection. Our objective in this study is to examine the psychometric properties of the Chinese Version of SIRS-2 using a simulation design (study one) and known-groups comparison design (study two) in China. There are two reasons for selecting these two designs: (a) Simulation designs is ideal for controlling many elements of the experimental design and provide the best basis for internal validity, but it possesses threats to external validity, as the use of simulators might decrease the generalizability of the results [17,18]; (b) known-groups comparison design is stronger for external validity, given their emphasis on real-world applications. Firstly, we examined the internal consistencies of the Chinese SIRS-2 subscales designed to detect malingering by calculating their Cronbach’s alpha coefficients. Second, we evaluated the discriminant validity of each subscale by examining Analysis of Variance (via effect sizes). Finally, we sought to determine which measures were most effective (i.e., sensitivity and negative predictive power, NPP) for evaluation of malingering.

## Methods

### Participants

#### Study one using simulation design

Eighty undergraduate students were recruited through advertisements placed in three University campuses of Hunan Province in Changsha City. Subjects were required to have no language or hearing impediments and no psychiatric history. All subjects were 18 years of age or more.

In addition, 40 consecutive inpatients with mental disorders were recruited from July 1 to December 30 2011 in the second Xiangya hospital of Central South University in Changsha, if they met the following inclusion criteria:1) patients diagnosed with mental disorders by two psychiatrists according to Diagnostic and Statistical Manual of Mental Disorders, fourth edition (DSM-IV); 2) understand scale content (We examined the subjects with a Structured Interview, and we used this procedure for every participant: first, we read out the question; then the participant would be asked to narrate the meaning of the question, if right, he could answer the question. If not, we would explain the meaning of the question to him); 3) must be age 18 or older. Diagnoses for the 40 psychiatric patients were mostly Axis I disorders: schizophrenia (55%), mood disorders (30%), substance dependence (2.5%), and stress-related disorders (2.5%).

#### Study two using known-groups comparison

One hundred subjects in forensic setting participated in known-groups comparison; they were recruited from two forensic psychiatric centers of the second Xiangya Hospital of the Central South University and the Rongjun Hospital. Subjects were screened to ensure that they were of age above 18 years old, and could understand scale content.

### Measurement

#### Chinese version of SIRS-2

The Chinese version of SIRS-2 was provided by Professor Tam, Wai -Cheong Carl, one of the investigators in this study, who had translated the SIRS-2 following a rigorous translation method. The 172-item Chinese Version of SIRS-2 is a structured interview designed to detect various exaggerated response styles. Each scale provides four classifications: honest, indeterminate, probable faking, and definite faking. The item score of Chinese Version of SIRS-2 ranges from 0 to 2 (0 = no, 1 = yes, 2 = unbearable), with higher scores suggesting feigned symptoms. The Chinese Version of SIRS -2 was scored using the criteria described by Rogers (i.e., at least one subscale in the definite malingering range and/or three subscales in the probable range) [19].

#### MMPI-2 Chinese version

MMPI-2 has been translated into Chinese and published by the Chinese University of Hong Kong with Hong Kong and China norms [20]. The MMPI-2 Chinese version has demonstrated excellent psychometric properties [20,21] and has been used in malingering detection in a sample of university students in Taiwan [22]. The F (Infrequency) subscale, L (Lie) and K (Correction) subscales of the MMPI-2 were regarded as the validity scales for the assessment of response styles systematically and empirically [23]. The F subscale performed well in the prediction on of the malingering. F subscale is associated with most fake-bad indicators, particularly those assessing exaggerated psychopathology. A lower score in K subscale was associated with exaggerated symptoms of psychosis. Other indexes and scales, including PAI and MCMI-III, were developed over the long life of MMPI to assess malingering. However there is no Chinese version for these scales, so we decided to use the MMPI-2 Chinese version in this study.

### Procedure

In study one, undergraduate students were randomly assigned to two different subgroups: honest students group (HS group, n = 40) and simulator students group (SS group, n = 40). HS group as well as the honest inpatients (HP group, n = 40) were asked to respond honestly in the interview. SS group subjects were asked to feign mental illness in the interview and they were given information about the common symptoms of mental disorders.

In study two, forensic experts interviewed 100 forensic subjects and reviewed the information from police as well as the history of the subjects. One hundred forensic subjects were divided into two groups according to the assessment of the forensic experts: forensic malingering group (FM group, n = 20), and forensic honest group (FH group, n = 80). One hundred forensic subjects in study two were asked to respond honestly in the interview. The Chinese Version of SIRS-2 was administered to all subjects in study one and two. At the same time the Chinese Version of MMPI-2 was administered to each participant from universities (HS + SS groups). All interviewers were trained in the use of the SIRS-2 according to the recommendations in the SIRS Manual [24]. All participants provided written informed consent to participate in this research. The study protocol was approved by the Second Xiangya Hospital medical ethics review committee.

### Data analysis

All statistical analyses were carried out using SPSS 17.0 Software for Windows. Correlation analyses were used to explore the correlations between the SIRS-2 Chinese version and MMPI-2 Chinese version to examine convergent and divergent validity of detection strategies, especially with the MMPI-2 indicators of fake-bad, defensiveness, and response consistency. For the discriminability of this scale, we used Analysis of Variance to examine the differences among honest students, simulators students and honest inpatients on the SIRS-2 in study one, and used analysis of independent t-test to examine the differences between forensic malingering group and forensic honest group on the SIRS-2 in study two. Magnitude of differences between individual groups was characterized by Cohen’s *d* effect size estimates. Classification analysis of different groups was used to explore the validity (predictive accuracy) of the SIRS-2 Chinese version, for example, sensitivity (i.e., proportion of malingerers accurately identified by the measure) and specificity rates (i.e., proportion of people accurately classified as responding honestly). The significance level was set at p < .05.

Cronbach’s alpha coefficients were calculated for eleven subscales (six primary subscales, four supplementary subscales and one classification subscale) of the SIRS-2 Chinese version (Selectivity of Symptoms(SEL) and Severity of Symptoms(SEV) subscales combine responses from the Blatant Symptoms(BL) and Subtle Symptoms(SU) subscales were therefore not analyzed).

## Results

### Reliability

For internal consistency of the Chinese Version of SIRS-2, Cronbach’s alpha coefficients were calculated in 220 participants (Table 1). The Cronbach’s alpha coefficients range from 0.66 to 0.92. Except for the subscales of direct appraisal of honesty, the Cronbach’s alpha coefficients of the various subscales of the Chinese Version of SIRS-2 were compatible with those of the original SIRS-2 cited in the Manual.

**Table 1:** Cronbach’s alphas for the SIRS-2(CV) subscales

**SIRS-2(from Manual)SIRS-2(CV)**			**SIRS-2(fromManual)SIRS-2(CV)**		
Primary scales			Supplementary scales		
RS	0.82	0.80	DA	0.50	0.79
SC	0.76	0.81	DS	0.77	0.84
IA	0.85	0.83	IF	0.92	0.89
BL	0.93	0.91	OS	0.82	
SU	0.84	0.91			0.81
RO	0.84	0.74			
Classification scale					
RS-Total	0.83	0.86			

Note. For Primary Scales, RS = Rare Symptoms, SC = Symptom Combinations, IA = Improbable or Absurd Symptoms, BL = Blatant Symptoms, SU = Subtle Symptoms, RO = Reported versus Observed Symptoms (RO), RS-Total = Rare Symptoms Total. For Supplementary Scales, DA = Direct Appraisal of Honesty, DS = Defensive Symptoms, IF = Improbable Failure (IF), OS = Overly Specified Symptoms.

### Criterion validity

Criterion validity of malingering measured by the Chinese Version SIRS-2 was tested in 80 undergraduates (HS group and SS group) against the scores on the MMPI-2. For convergent validity, scores on the primary scales of the Chinese Version of SIRS-2 were correlated positively well with the malingering ratings on MMPI-2, with the Infrequency dimension showing moderate correlation (r = 0.282-0.481, p < 0.05). For discriminant validity, the scores on the primary scales the Chinese Version of SIRS-2 were correlated negatively with the MMPI-2 indicators of Fake-bad (r = -0.255 ~ -0.519, p < 0.05), and Defensiveness (r = -0.205 ~ 0.391, p < 0.05). Table 2 shows the correlations of Primary Scales of SIRS-2 Chinese Version with the MMPI-2 validity scales.

**Table 2:** Correlations of the SIRS-2(CV) primary scales with the MMPI-2 validity scales (n = 80)

	**RS**	**SC**	**IA**	**BL**	**SU**	**SEL**	**SEV**	**RO**
Convergent validity								
F	0.284*	0.282*	0.227*	0.361**	0.351**	0.354**	0.481**	0.297**
Discriminant validity								
L	-0.284**	-0.300**	-0.255**	-0.420**	-0.326**	-0.413**	-0.519**	-0.330**
K	-0.296*	-0.239*	-0.229*	-0.314**	-0.205*	-0.227*	-0.391*	-0.232*

Note. For SIRS-2(CV) Primary Scales, RS = Rare Symptom Combinations, IA = Improbable or Absurd Symptoms, BL = Blatant Symptoms, SU = Subtle Symptoms, SEL = Selectivity of Symptoms, SEV = Severity of Symptoms, RO = Reported versus Observed Symptoms. For MMPI-2 validity scales, F = Infrequency, L = Lie, K = Correction. *p < 0.05. ** p < 0.01.

### Discriminant validity

In Study one, using simulation design, there were significant differences (e.g., RS, F(118) = 191.91, p < 0.05) in the three groups (HS, SS and HP group) of subjects in the scores of the eight primary subscales except three subscales (DA, Direct Appraisal of Honesty, DS, Defensive Symptoms, OS, Overly Specified Symptoms). For the total scores of Chinese version of SIRS-2, the SS group had the highest scores, followed by the HP group, the HS group had the lowest scores (Table 3). In Study two, using known-groups comparison, the FH group scored significantly lower than the FM group on the Chinese version of SIRS-2 subscales (e.g., RS, t(98) = -7.81, p < 0.05) (Table 4).

**Table 3:** Means and standard deviations of the Chinese version of SIRS-2 scales for simulation design sample

	**Simulator students**	**Honest inpatients**	**Honest students**	**F value**	**P value**
	**(n = 40)**	**(n = 40)**	**(n = 40)**		
	**Mean(SD)**	**Mean(SD)**	**Mean(SD)**		
Primary scales					
RS	10.23 (3.29)	2.38 (1.90)	0.73 (1.30)	191.91	p < 0.05
SC	11.73 (3.51)	4.33 (2.99)	1.05 (1.22)	188.47	p < 0.05
IA	9.33 (3.12)	1.28 (1.12)	0.80 (0.94)	232.92	p < 0.05
BL	18.48 (6.39)	8.73 (4.06)	0.55 (1.18)	164.47	p < 0.05
SU	13.85 (3.67)	8.05 (2.68)	2.15 (2.18)	161.77	p < 0.05
SEL	18.40 (7.27)	11.03 (3.53)	3.28 (2.52)	95.87	p < 0.05
SEV	6.80 (4.73)	3.08 (2.76)	0.05 (0.22)	45.68	p < 0.05
RO	6.10 (3.42)	0.43 (1.11)	0.33 (0.80)	96.83	p < 0.05
Supplementary scales					
DA	5.43 (2.50)	3.05 (1.80)	2.45 (1.40)	26.12	p > 0.05
DS	24.23 (5.77)	20.80 (6.33)	17.60 (4.07)	14.67	p > 0.05
IF	10.45 (5.84)	1.80 (2.64)	0.20 (0.91)	87.16	p < 0.05
OS	6.80 (3.04)	1.33 (1.44)	0.28 (0.60)	126.22	p > 0.05
INC	4.95 (4.45)	0.48 (1.59)	0.00 (0.00)	40.13	p < 0.05

Note. For Primary Scales, RS = Rare Symptoms, SC = Symptom Combinations, IA = Improbable or Absurd Symptoms, BL = Blatant Symptoms, SU = Subtle Symptoms, SEL = Selectivity of Symptoms, SEV = Severity of Symptoms, RO = Reported versus Observed Symptoms. For Supplementary Scales, DA = Direct Appraisal of Honesty, DS = Defensive Symptoms. IF = Improbable Failure (IF). OS = Overly Specified Symptoms. INC = Inconsistency of Symptoms.

**Table 4:** Means and standard deviations of the Chinese version of SIRS-2 scales for known-group design sample

	**Forensic malingering group (n = 20) Mean(SD)**	**Forensic honest group (n = 80) Mean(SD)**	**T value**	**P value**
Primary scales				
RS	7.40(1.73)	2.80 (2.58)	-7.81	p < 0.05
SC	6.75(1.12)	4.64 (3.25)	4.79	p < 0.05
IA	4.70(1.26)	1.43 (1.77)	7.78	p < 0.05
BL	13.80(1.99)	8.43 (5.07)	7.46	p < 0.05
SU	15.00(2.83)	7.85 (2.90)	9.92	p < 0.05
SEL	15.95(1.93)	9.48 (3.42)	11.23	p < 0.05
SEV	9.70(1.78)	3.34 (3.11)	12.04	p < 0.05
RO	5.05(1.43)	0.46 (1.21)	14.60	p < 0.05
Supplementary scales				
DA	5.45(1.91)	4.60 (2.47)	1.43	p > 0.05
DS	23.70(2.62)	21.99 (3.83)	1.89	p > 0.05
IF	10.40(3.62)	1.54 (2.41)	13.2	p < 0.05
OS	4.05(2.46)	3.06 (1.18)	1.75	p > 0.05
INC	5.65(0.67)	3.90 (2.41)	5.67	p < 0.05

Note. For Primary Scales, RS = Rare Symptoms, SC = Symptom Combinations, IA = Improbable or Absurd Symptoms, BL = Blatant Symptoms, SU = Subtle Symptoms, SEL = Selectivity of Symptoms, SEV = Severity of Symptoms, RO = Reported versus Observed Symptoms. For Supplementary Scales, DA = Direct Appraisal of Honesty, DS = Defensive Symptoms. IF = Improbable Failure (IF). OS = Overly Specified Symptoms. INC = Inconsistency of Symptoms.

The effect sizes of the subscales between SS group and HS group, SS group and HP group, FM group and FH group were shown in Table 5. As the table indicates, all the Cohen’*d* reflected significant differences between the group means (p < 0.05). The effect sizes of the Primary Scales for feigning (SS group and FM group) versus honest (HS group, HP group and FH group) samples of the original SIRS-2 cited in the Manual are also listed in the table for easy comparison. The mean effect sizes for three groups’ comparisons ranged from 1.79 to 1.80, which were compatible to the mean effect size of 2.08 of the validation samples cited in the Manual.

**Table 5:** Effect sizes (Cohen’s d) of the Chinese version of SIRS-2 scales among the participant groups

	**Honest inpatients vs. simulated students**	**Simulated students vs. honest students**	**Forensic malingering vs.forensic honest**	**SIRS-2 validation values (from Manual)**
Primary scales				
RS	1.62	2.16	1.91	2.04
SC	1.88	1.57	1.69	1.75
I A	1.06	1.78	1.44	1.80
BL	2.04	2.11	2.09	2.49
SU	1.83	1.38	1.55	2.12
SEL	2.14	2.06	2.10	2.37
SEV	2.15	1.62	1.82	1.95
RO	1.78	1.65	1.70	2.12
Supplementary scales				
DA	1.12	1.37	1.27	2.08
DS	0.88	0.44	0.62	NA
IF	0.48	0.32	0.39	NA
OS	1.00	1.03	1.02	NA
INC	1.06	1.00	1.03	NA

*Note*. For Primary Scales, RS = Rare Symptoms, SC = Symptom Combinations, IA = Improbable or Absurd Symptoms, BL = Blatant Symptoms, SU = Subtle Symptoms, SEL = Selectivity of Symptoms, SEV = Severity of Symptoms, RO = Reported versus Observed Symptoms. For Supplementary Scales, DA = Direct Appraisal of Honesty, DS = Defensive Symptoms. IF = Improbable Failure (IF). OS = Overly Specified Symptoms. INC = Inconsistency of Symptoms.

### Discriminant analyses of different groups

In study one, HP + HS (honest group) and SS (feign group) and HS group were used as the two groups to calculate the sensitivity and specificity of the Chinese version of SIRS-2. The overall hit rate for detecting malingering of approximately 20% was obtained when classifying HS group, HP group and SS group in simulation design. However, rates were increased to approximately 85% when classifying FM group and FH group in known-groups comparison.

The classification results of simulation design sample are shown in Table 6a. The sensitivity and specificity were 0.20 and 1.00 respectively, while the positive predictive power and negative predictive power were 1.00 and 0.58 respectively. In study 2, the FM group and FH group were used as the two groups to calculate the sensitivity and specificity of the Chinese version of SIRS-2. The classification results of known-group design sample are shown in Table 6b. The sensitivity and specificity were 0.85 and 1.00 respectively, while the positive predictive power and negative predictive power were 1.00 and 0.72 respectively. The corresponding values for the sensitivity and specificity of the Chinese version of SIRS-2 for clinical sample described in the Manual were 0.80 and 0.975 respectively, while both the positive predictive power and negative predictive power were 0.91.

**Table 6:** Classification results of the SIRS-2(CV)

**a. For the simulation design sample**		
	Predicted group membership	
		FFeigning others a
Actual group		
Feigning	24	16
Honest	0	80
**b. For the known-group design sample**		
	Predicted group membership	
		Feigning others a
Actual group		
Feigning	17	3
Honest	0	120

Note. a Others include indeterminate-evaluate, indeterminate-general, disengagement, and genuine responding.

## Discussion

The results of this study showed good reliability and validity of the Chinese version of SIRS-2. In our current two studies, we evaluated the internal consistency of Chinese version of SIRS-2, its cronbach’s alpha coefficients, these results converged with those of Rogers research to suggest that the primary scales had good internal consistency [25].

The SIRS-2 appears to be a promising screening instrument for malingering. However, SIRS-2 are not used for the definitive determination of malingering or feigned mental disorders, that is to say, there is need for a full evaluation of feigning together with other clinical data. Results from study one indicated a high level of discrimination for the primary scales between SS group and HS group and HP group, with the exception of the subscales of DA, DS, and OS. One expected finding was that the total scores of the SIRS for the feigners (SS group and FM group) were higher than those for the honest responders (HP group and HS group). The purpose of the SIRS-2 primary scales was to distinguish between feigned and genuine presentations of mental disorders [26]. Results of the current study generally supported this conclusion, with four subscales (BL, SU, SEL, and SEV) based on detection strategies in the amplified response category showing more effective identifying the malingerers than those subscales that relied on inconsistent response category. Overall, the effect sizes in our studies were moderate. In our study, sensitivity rates were most impressive in the study of forensic sample. And the sensitivity rates in detecting malingering among this sample of forensic subjects were superior to the sensitivity rates described by Rogers et al., [27] (80% for Rogers et al. study vs. 85% for our study). The sensitivity rates for detecting malingering among the University students was substantially lower than for the forensic sample (60% vs. 85%). One possibility for the decreased sensitivity among university students asked to feign symptoms is that they might not understand the symptoms of mental illness very well. Research shows that financial compensation does affect patients’ performance in clinical contexts [28,29]. Thus, it is likely the absence of significant financial incentives for the participants in the present study influenced their performance.

Since the MMPI-2 is the most popular and well- researched objective clinical assessment instrument [30], the construct validity of the Chinese version of SIRS-2 was examined by using MMPI-2. The SIRS primary scales produced correlations with MMPI-2 feigning scales with inverse relations with its scales of Defensiveness. The eight primary scales of the Chinese version of SIRS-2 which were designed to identify feigned mental disorders performed well in this study, often exhibiting the significant positively correlation with F subscale, and exhibiting the significant negatively correlation with K/L of the MMPI-2. The correlation between the MMPI validity scales and the malingering test are statistically significant but very low in order to claim that there is enough covariance between them to be considered measures of the same criteria. This finding is not enough to identify two different measures as meaningfully convergent on the same variable.

## Conclusion

The present study found that the Chinese version of the SIRS-2 had good reliability and validity in detecting malingering and would be a suitable tool to use in China.

## Abbreviations

SIRS-2: Structured interview of reported symptoms-2; MMPI-2: Minnesota multiphasic personality inventory-2; DSM-IV: Diagnostic and statistical manual, fourth edition; PAI: Personality assessment inventory; MCMI-III: Miller clinical multiphasic inventor-III; RS: Rare symptoms; SC: Symptom combinations; IA: Improbable or absurd symptoms; RO: Reported versus observed symptoms; BL: Blatant symptoms; SU: Subtle symptoms; SEL: Selectivity of symptoms; SEV: Severity of symptoms; DA: Direct appraisal of honesty; DS: Defensive symptoms; IF: Improbable failure; OS: Overly specified symptoms; INC: Inconsistency of symptoms; RS-total: Rare symptoms total; MT index: Modified total index; SS index: Supplementary scale index; F: Infrequency; L: Lie; K: Correction; HS group: Honest students group; SS group: Simulator students group; HP group: Honest patients group; FH group: Forensic honest group; FM group: Forensic malingering group; NPP: Negative predictive power.

## Competing interests

All authors declare that they have no conflicts of interests.

## Authors’ contributions

ZL and TW-CC designed the study. CL, PW, LC, HA, YW and GW managed the data collection. CL, HZ and TW-CC undertook the statistical analysis. CL and HC wrote the first draft of the manuscript. We all contributed to and approved the final manuscript. All authors read and approved the final manuscript.

## Pre-publication history

The pre-publication history for this paper can be accessed here:

http://www.biomedcentral.com/1471-244X/13/254/prepub
